# Author Correction: FGL2 promotes tumor progression in the CNS by suppressing CD103^+^ dendritic cell differentiation

**DOI:** 10.1038/s41467-019-08770-5

**Published:** 2019-02-15

**Authors:** Jun Yan, Qingnan Zhao, Konrad Gabrusiewicz, Ling-Yuan Kong, Xueqing Xia, Jian Wang, Martina Ott, Jingda Xu, R. Eric Davis, Longfei Huo, Ganesh Rao, Shao-Cong Sun, Stephanie S. Watowich, Amy B. Heimberger, Shulin Li

**Affiliations:** 1 Center for Brain Disorders Research, Capital Medical University, Beijing, 100069 China; 2 Beijing Institute for Brain Disorders, Beijing, 100069 China; 3 Department of Pediatrics–Research, The University of Texas MD Anderson Cancer Center, Houston, TX 77030 USA; 4 Department of Neurosurgery, The University of Texas MD Anderson Cancer Center, Houston, TX 77030 USA; 5 Department of Biostatistics, The University of Texas MD Anderson Cancer Center, Houston, TX 77030 USA; 6 Department of Lymphoma and Myeloma, The University of Texas MD Anderson Cancer Center, Houston, TX 77030 USA; 7 Department of Immunology, The University of Texas MD Anderson Cancer Center, Houston, TX 77030 USA

Correction to: *Nature Communications*  10.1038/s41467-018-08271-x, published online 25 Jan 2019.

The original version of this Article contained errors in the author affiliations.

Qingnan Zhao, Xueqing Xia, Longfei Huo and Shulin Li were incorrectly associated with Beijing Institute for Brain Disorders, 100069, Beijing, China.

This has now been corrected in both the PDF and HTML versions of the Article.

