# Supplementary material for: FGL2 promotes tumor progression in the CNS by suppressing CD103+ dendritic cell differentiation
Source: Nat Commun. 2019 Jan 25;10:448. doi: 10.1038/s41467-018-08271-x (PMC6347641; doi:10.1038/s41467-018-08271-x)
Supplement: Supplementary file 1 — Supplementary Information [file 41467_2018_8271_MOESM1_ESM.pdf]

Supplementary Information:

FGL2 promotes tumor progression in the CNS by suppressing CD103$^+$ dendritic cell differentiation

Yan et al.

Supplementary Figures and Legends

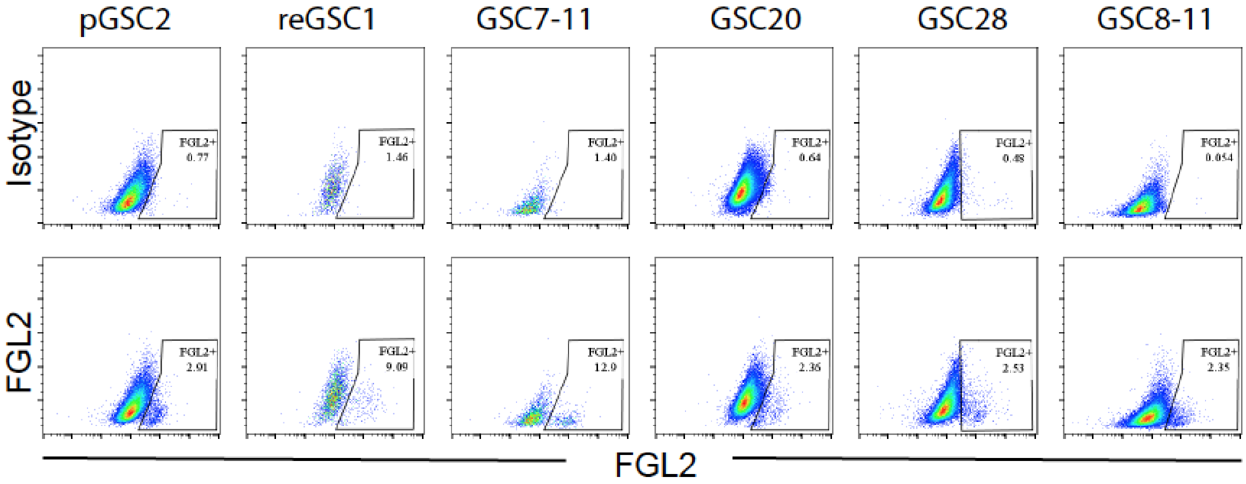

Supplementary Figure 1. FGL2 expression on cultured human glioma stem cells (GSCs). Cultured GSCs (pGSC2, reGSC1, GSC7-11, GSC20, GSC28, and GSC8-11) were pooled, stained with anti-FGL2 antibody, and detected by flow cytometry. Plots are representative of three independent experiments.

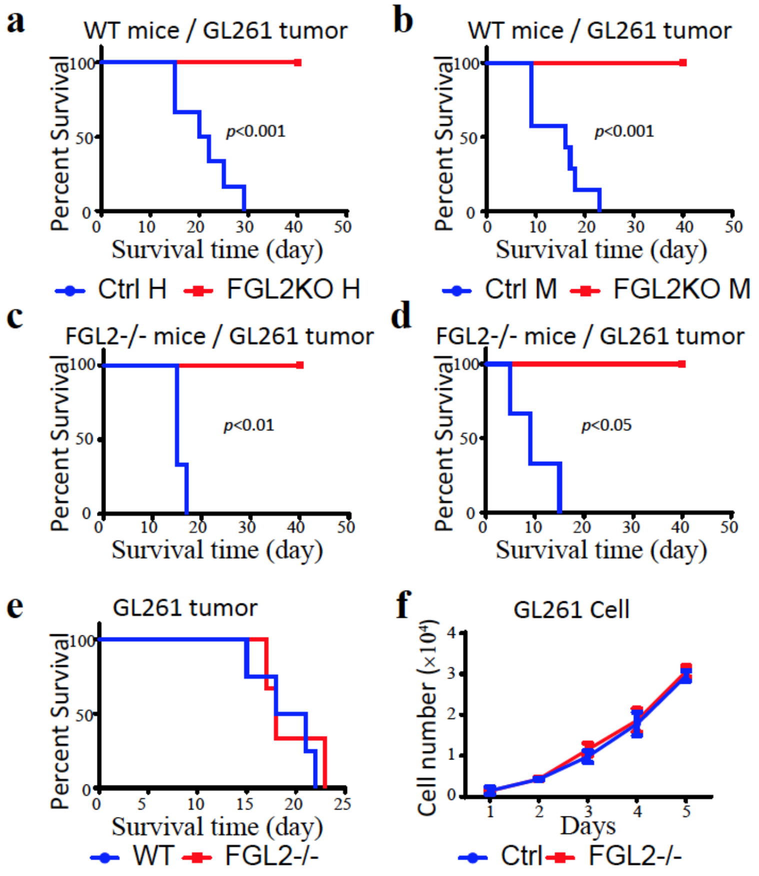

Supplementary Figure 2. Progression of *Ctrl* and *FGL2KO* tumor cells *in vivo* and *in vitro*. a-b, Survival analyses for immunocompetent *wild-type (WT)* C57BL/6 mice inoculated with control (GL261-*Ctrl*) or knockout (GL261-*FGL2KO*) tumor cells in high (H) numbers (2.5×10^5 cells, **a**) or maximal (M) numbers (1×10^6 cells, **b**) (n=6~7/ group). **c-d**, Survival analyses for *FGL2*$^{-/-}$ mice inoculated with GL261-*Ctrl* or GL261-*FGL2KO* tumor cells in high numbers (2.5×10^5 cells, **c**) or maximal numbers (1×10^6 cells, **d**) (n=6). **e**, Survival analysis for C57BL/6 *WT* and *FGL2*$^{-/-}$ mice bearing an intracranial GL261 tumor (n=5~9/group). **f**, Growth of GL261-*Ctrl* and GL261-*FGL2KO* cells determined by MTT cell proliferation assay. Data represent the mean ± S.D. of three independent experiments. The survival curves were analyzed by Kaplan-Meier analysis and the log-rank test was used to compare overall survival between groups.

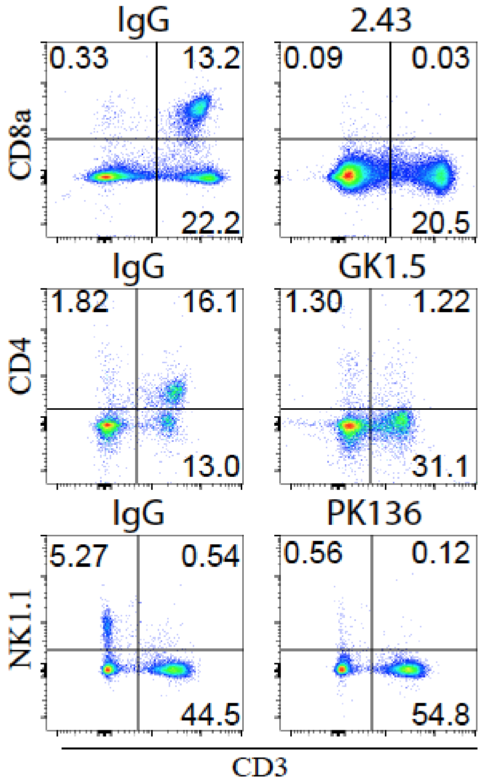

Supplementary Figure 3. **Depletion efficacy of CD4^{+} T, CD8^{+} T, or natural killer (NK) cells in mice.** Cells from spleens of mice subjected to antibody-mediated depletion of CD4^{+} T, CD8^{+} T, or NK cells or control antibody IgG were subjected to staining and analyzed by flow cytometry.

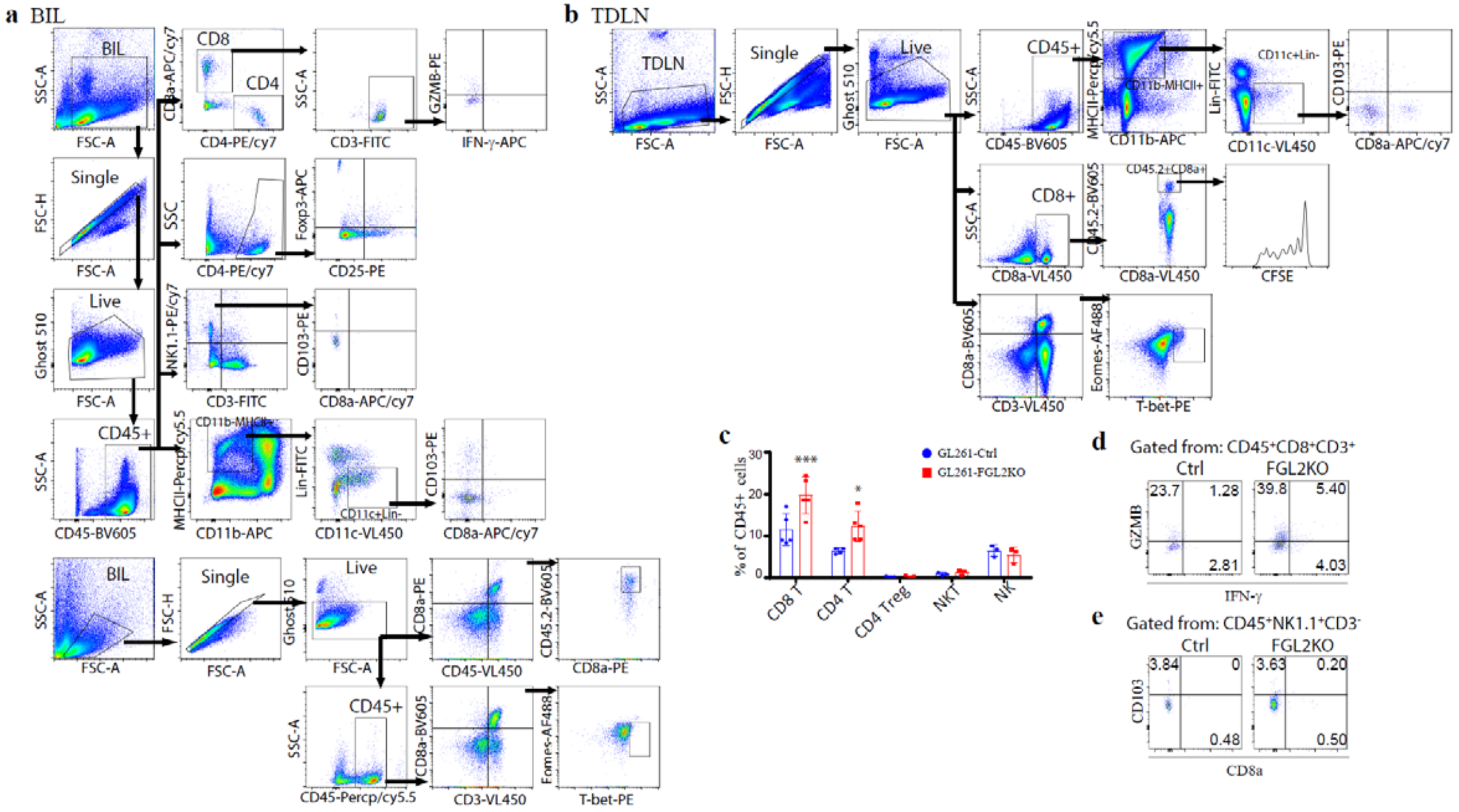

Supplementary Figure 4. ***FGL2KO* tumors exhibited accumulation of CD8^{+} T cells in brains and TDLNs. a-b**, The FACS gating strategy for identification of CD4^{+} T, CD8^{+} T, CD4^{+} Treg, NK, DC, and OT-I cells in the brain or in TDLNs is shown (correspond to Figure 4a, 4e, 4f, Supplementary Figure 4c-4e,), as well as the results showing expression of CD8a and CD103 on NK cells and DCs. Brains or TDLNs from *FGL2KO* and *wild-type (WT)* tumor–bearing mice were collected; single-cell suspensions of isolated brain-infiltrating leukocytes or TDLNs were subjected to antibody staining and FACS analysis. Lineage^{-}, CD3^{-}NK1.1^{-}Gr1^{-}Ter119^{-}. **c**, CD4^{+} T, CD8^{+} T, CD4+ Treg, NKT, and NK cells in the tumor microenvironment in mice were quantified 7 days after tumor inoculation (n=3~5/group). Data are presented as the mean ± S.D. and were analyzed by unpaired *t*-test. *P<0.05, ***P<0.001 vs GL261-*Ctrl*. **d**, IFNγ and/or granzyme B (GZMB) expression in CD8^{+} T cells from brains of control (GL261-*Ctrl*) or *FGL2* knockout (GL261-*FGL2KO*) tumor-bearing mice. **e**, CD8a and/or CD103 expression in NK cells from brains of control (GL261-*Ctrl*) or knockout (GL261-*FGL2KO*) tumor-bearing mice.

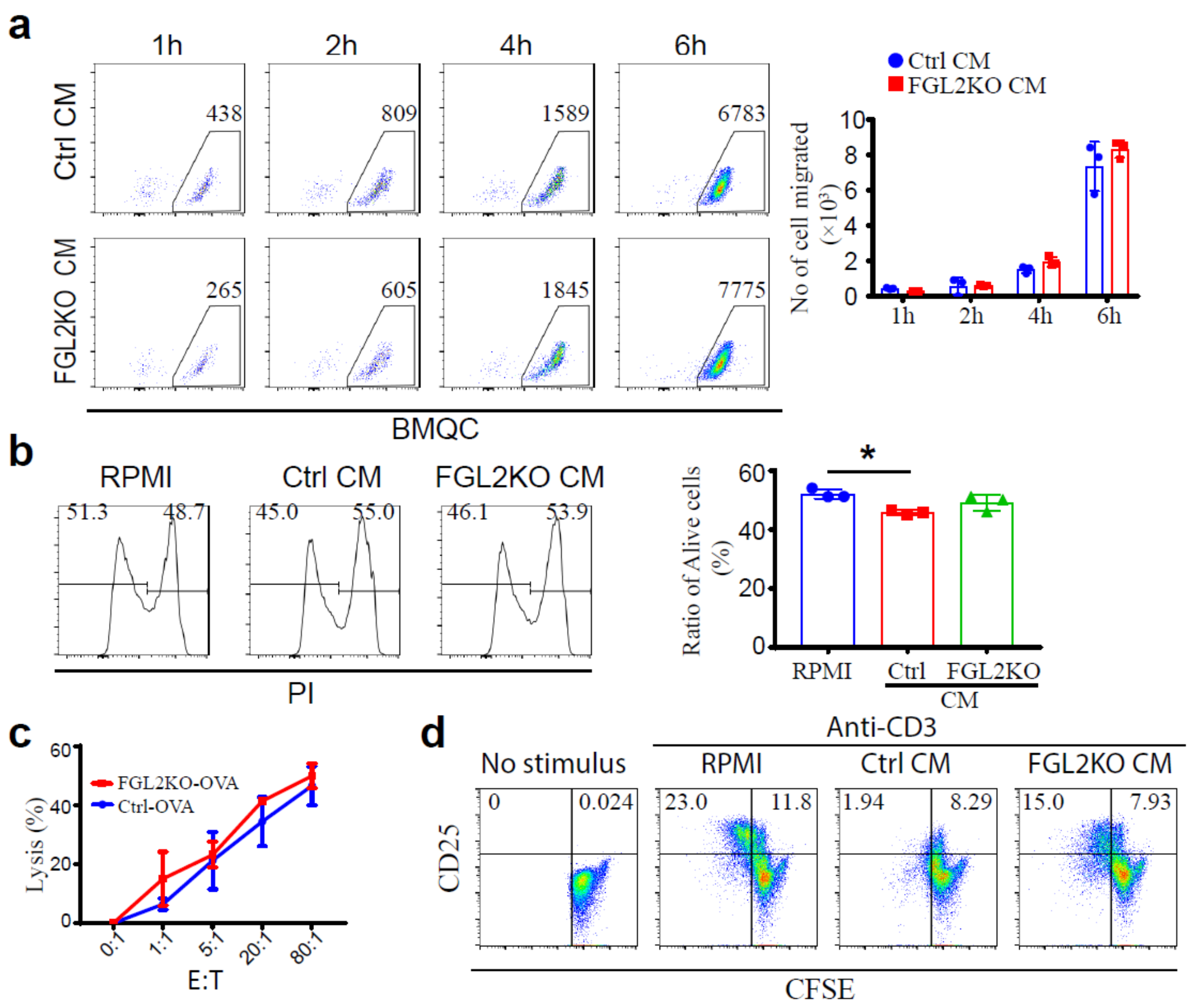

Supplementary Figure 5. **The effect of FGL2 on migration, survival, effector function, and activation of CD8⁺ T cells. a**, Cell migration of OVA peptide–activated CD8⁺ T cells (OT-I) induced by conditioned medium (CM) from control (GL261-*Ctrl*-*OVA*) or knockout (GL261-*FGL2KO*-*OVA*) tumor cells (n=3). The numbers of migrated BMQC⁺ OT-I cells in the bottom chambers were detected via FACS. Data are presented as the mean ± S.D. of three independent experiments and were analyzed by two-way ANOVA. **b**, Survival of activated OT-I cells cultured in the indicated CM for 5 days (n=3). The PI⁻ live cell population was detected via FACS. Data are presented as the mean ± S.D. of three independent experiments and were analyzed by one-way ANOVA. *P<0.05. **c**, Relative killing by OT-I cells of GL261-*Ctrl*-*OVA* and GL261-*FGL2KO*-*OVA* tumor cells after 6 h co-incubation. Data are presented as the mean ± S.D. of three independent experiments. **d**, Effect of CM from GL261-*Ctrl* or GL261-*FGL2KO* tumor cells on CD8⁺ T-cell activation and proliferation induced by anti-CD3 *in vitro*. Plots are representative of three independent experiments.

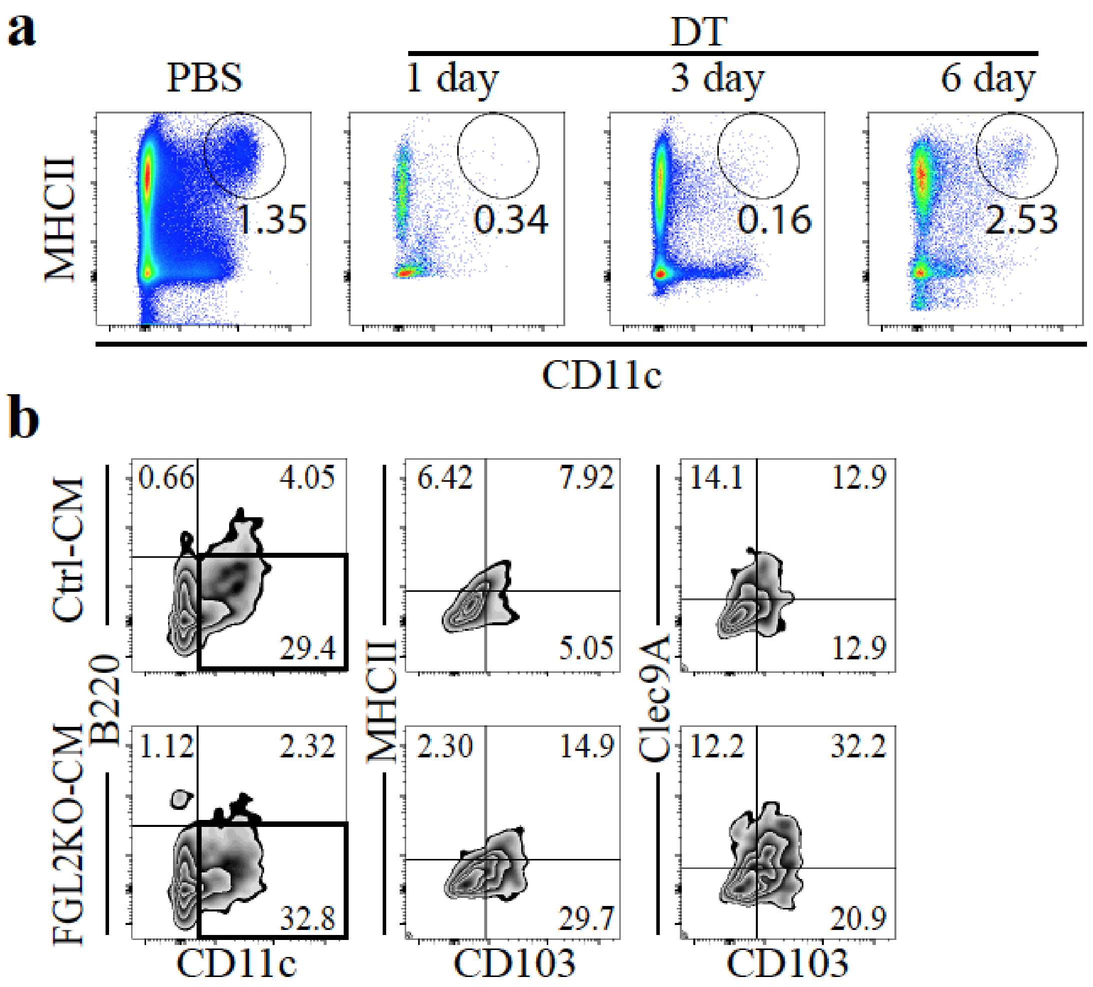

Supplementary Figure 6. **Flow cytometry plots of DCs population in CD11c-DTR mice and cultured bone marrow cells. a**, DCs in the spleens of *CD11c-DTR* mice at day 1, 3, and 6 post injection of diphtheria toxin (DT). Spleens were collected at each of those time points, and CD11c$^+$MHCII$^+$ cells were detected by flow cytometry. **b,** Bone marrow cells were cultured with conditioned medium (CM) from *Ctrl* or *FGL2KO* cells for 15 days, and non-attached cells were collected. CD11c$^+$B220$^-$ DCs were analyzed for CD103, Clec9A, and MHCII expression. Plots are representative of three independent experiments.

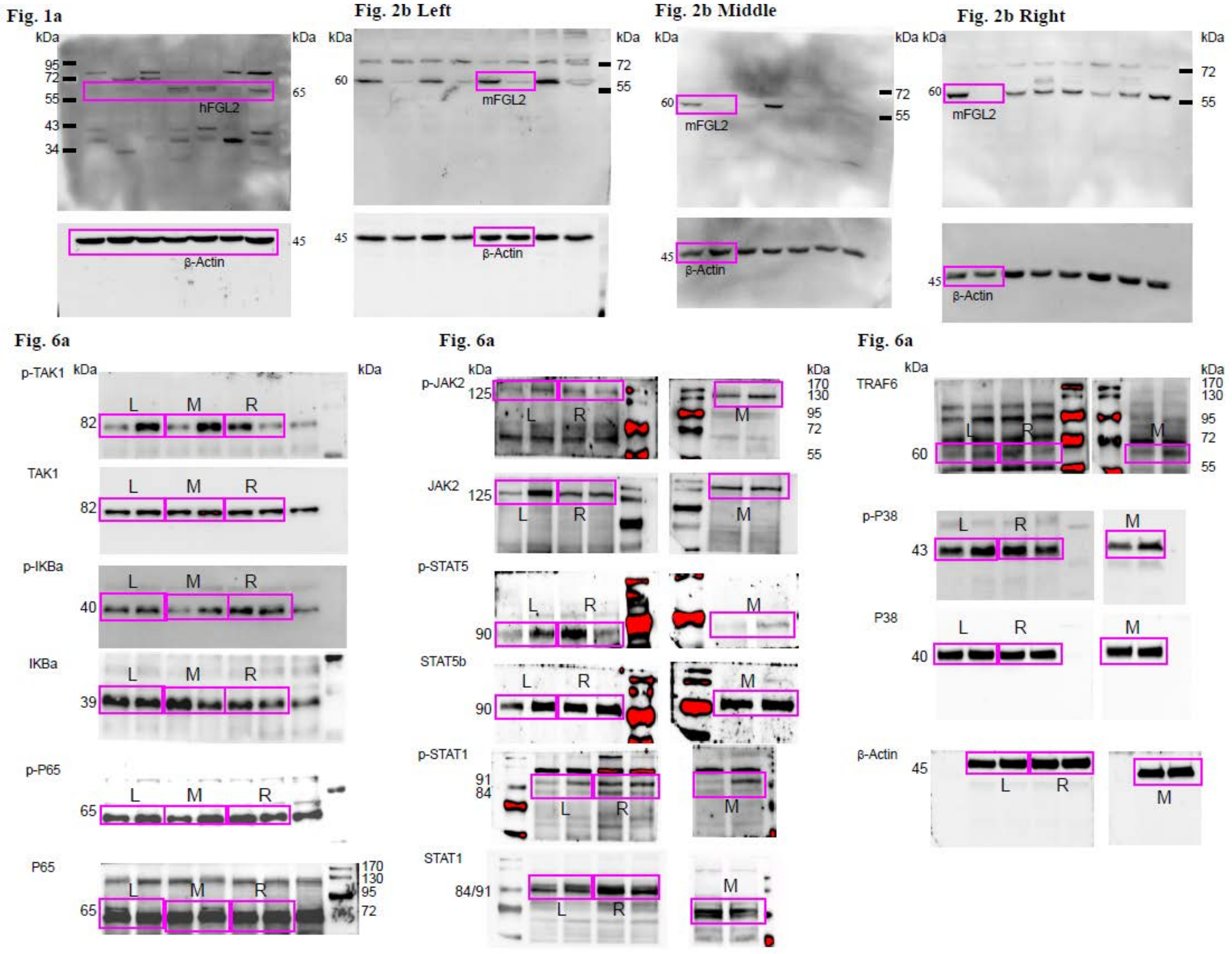

Supplementary Figure 7. Uncropped images of immunoblots for Figure 1a, 2b, 6a.

Gene	OS Assoc	HR (95% CI)	p value
FGL2	-	1.113 (0.991 - 1.25)	0.071
GM-CSF	Increased	0.854 (0.76 - 0.959)	0.008
CD8B	-	0.906 (0.811 - 1.011)	0.078
IFNG	-	0.931 (0.838 - 1.034)	0.182
$FGL2^{low}GM-CSF^{high}$ vs Others	Increased	0.695 (0.546 - 0.883)	0.003
$FGL2^{low}CD8B^{high}IFNG^{high}$ vs $FGL2^{high}CD8B^{low}IFNG^{low}$	Increased	0.468 (0.292 - 0.749)	0.00155

Supplementary Table 1. Associations between FGL2, GM-CSF, CD8B, and IFNG gene expressions and OS. Data on survival of GBM patients and gene mRNA expression data (n=401 patients) were downloaded and retrieved from the TCGA data portal ([http://www.cbioportal.org/public-portal/] Accessed between January 1, 2018, and March 15, 2018). The expression levels for all genes were categorized as high or low using median values as the cutoffs. Hazard ratios (HRs) with 95% confidence intervals (CIs) were reported, where a HR value <1 means increased overall survival (OS) and >1 means decreased OS.
