# Supplementary material for: FGL2 promotes tumor progression in the CNS by suppressing CD103+ dendritic cell differentiation
Source: Nat Commun. 2019 Jan 25;10:448. doi: 10.1038/s41467-018-08271-x (PMC6347641; doi:10.1038/s41467-018-08271-x)
Supplement: Supplementary file 3 — Reporting Summary [file 41467_2018_8271_MOESM3_ESM.pdf]

nature research

Corresponding author(s): Shulin Li, Amy B Heimberger

Reporting Summary

Nature Research wishes to improve the reproducibility of the work that we publish. This form provides structure for consistency and transparency in reporting. For further information on Nature Research policies, see Authors & Referees and the Editorial Policy Checklist.

Statistical parameters

When statistical analyses are reported, confirm that the following items are present in the relevant location (e.g. figure legend, table legend, main text, or Methods section).

n/a	Confirmed	
☐	☒	The exact sample size (*n*) for each experimental group/condition, given as a discrete number and unit of measurement
☐	☒	An indication of whether measurements were taken from distinct samples or whether the same sample was measured repeatedly
☐	☒	The statistical test(s) used AND whether they are one- or two-sided<br>*Only common tests should be described solely by name; describe more complex techniques in the Methods section.*
☒	☐	A description of all covariates tested
☐	☒	A description of any assumptions or corrections, such as tests of normality and adjustment for multiple comparisons
☐	☒	A full description of the statistics including central tendency (e.g. means) or other basic estimates (e.g. regression coefficient) AND variation (e.g. standard deviation) or associated estimates of uncertainty (e.g. confidence intervals)
☐	☒	For null hypothesis testing, the test statistic (e.g. *F*, *t*, *r*) with confidence intervals, effect sizes, degrees of freedom and *P* value noted<br>*Give P values as exact values whenever suitable.*
☒	☐	For Bayesian analysis, information on the choice of priors and Markov chain Monte Carlo settings
☒	☐	For hierarchical and complex designs, identification of the appropriate level for tests and full reporting of outcomes
☒	☐	Estimates of effect sizes (e.g. Cohen's *d*, Pearson's *r*), indicating how they were calculated
☐	☒	Clearly defined error bars<br>*State explicitly what error bars represent (e.g. SD, SE, CI)*

Our web collection on statistics for biologists may be useful.

Software and code

Policy information about availability of computer code

Data collection	No software used.
Data analysis	R package software (R Development Core Team, Version 3.3.2)

For manuscripts utilizing custom algorithms or software that are central to the research but not yet described in published literature, software must be made available to editors/reviewers upon request. We strongly encourage code deposition in a community repository (e.g. GitHub). See the Nature Research guidelines for submitting code & software for further information.

Data

Policy information about availability of data

All manuscripts must include a data availability statement. This statement should provide the following information, where applicable:

- Accession codes, unique identifiers, or web links for publicly available datasets
- A list of figures that have associated raw data
- A description of any restrictions on data availability

The authors declare that all data supporting the findings of this study are available within the paper and its Supplementary Information files or from the corresponding author upon reasonable request.

Field-specific reporting

Please select the best fit for your research. If you are not sure, read the appropriate sections before making your selection.

☒ Life sciences ☐ Behavioural & social sciences ☐ Ecological, evolutionary & environmental sciences

For a reference copy of the document with all sections, see nature.com/authors/policies/ReportingSummary-flat.pdf

Life sciences study design

All studies must disclose on these points even when the disclosure is negative.

Sample size	For the animal and cell line studies, sample sizes were decided based on the similar studies reported in the previous literature. Given 3-9 repeated or mice per group, we have 80% power to detect a difference in means between two groups in the range of 3.0-1.4*sd at a significance level of 0.05 using two-sample t-test. For the TCGA mRNA expression data analysis, we used all the data from the cohort (401 patients). Given 401 patients and about 80% event rate, we have 80% power to detect a log hazard ratio of 0.16 at a significance level of 0.05.
Data exclusions	We removed outliers if the data were not met the assumption for homogeneity of variances.
Replication	Three different cell lines were used for testing reproducibility of our findings. The findings were reproduced among these three cell lines.
Randomization	No randomization was conducted for the samples or animals in our study. No randomization was conducted for the animal studies. Same age and gender of mice were distributed into different groups.
Blinding	The investigator was blinded to cells for tumor implantation.

Reporting for specific materials, systems and methods

Materials & experimental systems

n/a	Involved in the study	
☒	☐	Unique biological materials
☐	☒	Antibodies
☐	☒	Eukaryotic cell lines
☒	☐	Palaeontology
☐	☒	Animals and other organisms
☐	☒	Human research participants

Methods

n/a	Involved in the study	
☒	☐	ChIP-seq
☐	☒	Flow cytometry
☒	☐	MRI-based neuroimaging

Antibodies

Antibodies used	anti-FGL2 antibody (1：100, H00010875; Novus Biologicals, Littleton, CO, USA), anti-mouse Alexa Fluor 647-conjugated antibody. Fab fragment anti-mouse IgG (15-007-003; Jackson ImmunoResearch Laboratories, Inc., West Grove, PA, USA), rabbit anti-CD31 antibody (bs-0468R, 1:100; Bioss Inc., Woburn, MA, USA), mouse anti-CD45 antibody (MAB1430, 10 μg/mL; R&D Systems), or Alexa Fluor 488-conjugated GFAP (53-9892, 5 ?g/mL; Thermo Fisher Scientific), Alexa Fluor 488-conjugated anti-mouse/rabbit antibody (1：300; Invitrogen).The antibodies were purchased from Cell Signaling Technology (Danvers, MA) or Santa Cruz Biotechnology (Dallas, TX): αTRAF6 (D21G3), αp-TAK1 (Ser412), αTAK1 (D94D7), αp-IKBa (14D4), αIKBa (L35A5), αp-P65 (93H1), αP65 (D14E12), αp-P38 (D3F9), αP38 (D13E1), αp-JAK2 (D15E2), αJAK2 (D2E12), αp-ST1 (D3B7), αST1 (B-9), αp-ST5 (C11C5), αST5b (G-2), and αGAPDH (D16H11).
Validation	Validations were performed by the providers.

Eukaryotic cell lines

Policy information about cell lines

Cell line source(s)	Glioblastoma (GBM) stem cell (GSC) lines (GSC7-2, GSC11, GSC20, GSC28, pGSC2, reGSC1, GSC7-11, and GSC8-11) were obtained through Dr. Frederick F. Lang5, and HMVEC-L cells were provided by Dr. Schadler Keri (both of MD Anderson). The mouse glioma GL261 cells were obtained from the National Cancer Institute (Rockville, MD, USA), and cortical HCN-1A neurons from ATCC (Manassas, VA, USA). The DBT cells were kindly provided by Dr. Leonid Metelitsa (Baylor College of Medicine, Houston, TX, USA).

Authentication	Cell lines were authenticated by the providers.
Mycoplasma contamination	Cells were treated with mycoplasma removal reagents before using.
Commonly misidentified lines (See ICLAC register)	N/A

Animals and other organisms

Policy information about studies involving animals; ARRIVE guidelines recommended for reporting animal research

Laboratory animals	C57BL/6J, BALB/c, CD45.1 (B6.SJL-Ptprca Pepcb/BoyJ, 002014), OT-I (C57BL/6-Tg(TcraTcrb)1100Mjb/J, 003831), NSG (NOD.Cg-Prkdcscid Il2rgtm1Wjl /SzJ, 005557), CD11c-DTR (B6.FVB-1700016L21RikTg(Itgax-DTR/EGFP)57Lan, 004509), and Batf3-/- (B6.129S(C)-Batf3tm1Kmm/J, 013755) mice were obtained from the Jackson Laboratory (Bar Harbor, ME, USA). FGL2-/- mice were a gift from Dr. Gary Levy2 (Toronto General Hospital/Research Institute, Toronto, ON, Canada). CD8-/- mice were generated in our lab. All mice were aged 6 to 8 weeks when the experimental procedures began. Both gender were used in experiments. Their respective genetic backgrounds are C57BL/6 background except NSG mice.
Wild animals	N/A
Field-collected samples	N/A

Human research participants

Policy information about studies involving human research participants

Population characteristics	Resected tumor tissues from newly diagnosed primary GBM patients were used in this study.
Recruitment	Sample collection was conducted under protocol #LAB03-0687, which was approved by the Institutional Review Board of The University of Texas MD Anderson Cancer Center, after written informed consent was obtained. Patients' tumors were graded by a neuropathologist according to the World Health Organization classification.

Flow Cytometry

Plots

Confirm that:

☒ The axis labels state the marker and fluorochrome used (e.g. CD4-FITC).

☒ The axis scales are clearly visible. Include numbers along axes only for bottom left plot of group (a 'group' is an analysis of identical markers).

☒ All plots are contour plots with outliers or pseudocolor plots.

☒ A numerical value for number of cells or percentage (with statistics) is provided.

Methodology

Sample preparation	Patient tumor tissues were minced and enzymatically digested to obtain single-cell suspensions for staining. Myelin debris was removed by using Myelin Isolation Beads (130-104-262; Miltenyi Biotec, Bergisch Gladbach, Germany). Mouse brain tissues and tumor-draining lymph nodes (TDLNs) were minced and enzymatically digested to obtain single-cell suspensions. Brain-infiltrating leukocytes were isolated according to a previously published protocol73. Briefly, each single-cell suspension was subjected to centrifugation through a 30% Percoll gradient at 7800g for 30 min. The leukocyte layer was collected and subjected to centrifugation on a discontinuous Ficoll-Paque Plus gradient to select and purify leukocytes.
Instrument	LSRFortessa Analyzer with 5 lasers and 18 colors.
Software	BD FACSDiva 8.0.1 and FlowJo V10
Cell population abundance	The purities of CD8+ T cells enriched by MojoSort Mouse CD8 T cell Isolation Kit were over 90% as detected by FACS. The purities of DCs isolated by an EasySep Mouse Pan-DC Enrichment Kit Immunomagnetic negative selection cell isolation kit were about 85% as determined by FACS.
Gating strategy	The Fluorescence Minus One Control, or FMO control were used to define the boundaries between positive and negative cell populations in mutiple fluorochromes panel.

☒ Tick this box to confirm that a figure exemplifying the gating strategy is provided in the Supplementary Information.
